# The effect of informal social support on the health of Chinese older adults: a cross-sectional study

**DOI:** 10.1186/s12889-023-15837-y

**Published:** 2023-05-30

**Authors:** Ciran Yang, Changli Jia, Shicheng Yin, Zongfu Mao, Dan Cui

**Affiliations:** 1 School of Public Health, Wuhan University, 115# Donghu Road, Wuhan, 430071 China; 2 Global Health Institute, Wuhan University, Wuhan, China; 3 School of Medicine and Health Managemen, Tongji Medical College of Huazhong University of Science and Technology, Wuhan, China

**Keywords:** Informal social support, The health of chinese older adults, The quality of well-being scale (QWB), Cross-sectional study, Econometric methods, Mechanism, Heterogeneity

## Abstract

**Background:**

This study aimed to assess the effect of informal social support (ISS) on the health of Chinese older adults, identify channels of the association between the two, and assess the magnitude of this effect in different groups of older adults.

**Methods:**

Based on the data from the 2018 China Longitudinal Aging Social Survey (CLASS), we first used both the Quality of Well-Being (QWB) scale and the analytic hierarchy process (AHP) method to construct the QWB score that can objectively measure the health status of Chinese older adults. Next, we conducted an econometric equation controlling for various high-dimensional fixed effects, estimated the effects using the Tobit model, and used various robustness check strategies and the propensity score matching (PSM) method to ensure reliability and deal with the potential endogeneity, respectively. Finally, we performed staging and grouping regression for mechanism and heterogeneity analysis.

**Results:**

The mean QWB score of Chinese older adults was 0.778. ISS has a significant positive effect on the health of older adults (*P* < 0.001), and there were similar patterns of findings for the effects of SE (*P* < 0.001), PSS (*P* < 0.001), and ES (*P* < 0.001). Additionally, the health promotion effect is higher in older adults who are male (*P* < 0.001), under the age of 80 (*P* < 0.001), with agricultural household registration (*P* < 0.001), or with high income (*P* < 0.001) than in the control group.

**Conclusion:**

ISS, including SE, PSS, and ES, had significant promotion effects on the health of older adults, especially on those who are male, under the age of 80, with agricultural household registration, or with high income. Meanwhile, these effects could be reflected through two channels: alleviating loneliness and improving the positive emotional status of older adults.

## Background

One of the critical issues facing industrialized countries is the global trend of an unprecedented and increasing population aging, which continues to present significant challenges and lead to severe consequences [1, 2]. China, in particular, is experiencing a rapid pace of population aging, more urgent and distinct than other countries like Japan and the United States [3, 4]. The results of the seventh national census from China Statistical Yearbook–2021 show that there are 264.02 million people aged 60 + in China, accounting for 18.70%, an increase of 5.44% points compared with 2010. Projections estimate that by 2030, China will enter a super-aged society, with the share of the population aged 65 + reaching 20% [5]. This indicates that China will be one of the countries in the world with the highest share of older adults [6], and the problems derived from the irreversible population aging trend will burden the whole society [7]. Thus, effectively handling the all-around challenges arising from population aging is a major practical problem that needs systematic solutions.

Older adults, considered a vulnerable group in social life, often face more health risks due to physiological decline and weakened activity ability [8, 9]. The health status of older adults affects their quality of life and the pension burden of the family and society. Improving the overall health level of older adults and exploring their potential human capital positively impacts their life quality, which is of great practical significance for reducing the pension burden and promoting social harmony and stability. To achieve this goal, the Chinese government has conducted institutional exploration and practical work. In November 2021, the CPC Central Committee and the Chinese State Council issued the “Opinions on Strengthening Aging Work in the New Era,” whose measures will further optimize critical institutional systems covering various fields and provide strong social support for promoting the health and development of older adults, which further illustrates that from invisible system construction to actual policy practices, government-led social support has always played a fundamental and persistent role in shaping the overall health status of older adults. However, we should also see the diverse connotations of social support. At least from the perspective of categorization, in addition to formal social support (FSS), such as government-led health services, medical security, and pension security, informal social support (ISS) has been accumulated and acted over the life course of older adults. Specifically, ISS is regarded as an individual’s perception or experience of being cared for and respected by others in social interaction, which shows that ISS, the classic and widely known concept, is distinct from the FSS [10, 11]. In China’s specific social context, whether from reality perception or academic discussion, the role of FSS in promoting the health of older adults has been widely recognized [12–14]. However, the role of ISS impacting the health of older adults has still been poorly analyzed in a clear theoretical and empirical way. For the Chinese society that is already in the aging stage and is still accelerating towards the super-aging process, we must not only focus on the utility of the FSS provided by the government but also identify and explore other factors that may promote the health of older adults [15]. Only in this way can we help to form a general and sustainable health maintenance and promotion system, which has certain theoretical inspiration and practical value for reducing the social and individual pension burden and relieving the impact of population aging.

Therefore, this study aims to explore the effect of ISS on the health of the Chinese older adults, hypothesizing that ISS plays a significant role in promoting their health, and can serve as a valuable supplement to government-led FSS. To test this hypothesis, we use data from the 2018 China Longevity Aging Social Survey (CLASS) to measure the current health status of Chinese older adults by constructing a multidimensional index and assess the effect of ISS on their health. Additionally, we conducted the mechanism analysis and heterogeneity analysis to enrich the research content and provide policy recommendations that can help improve the health of older adults and reduce the cost of older care based on empirical results.

## Methods

### Data sources

**Fig. 1:**
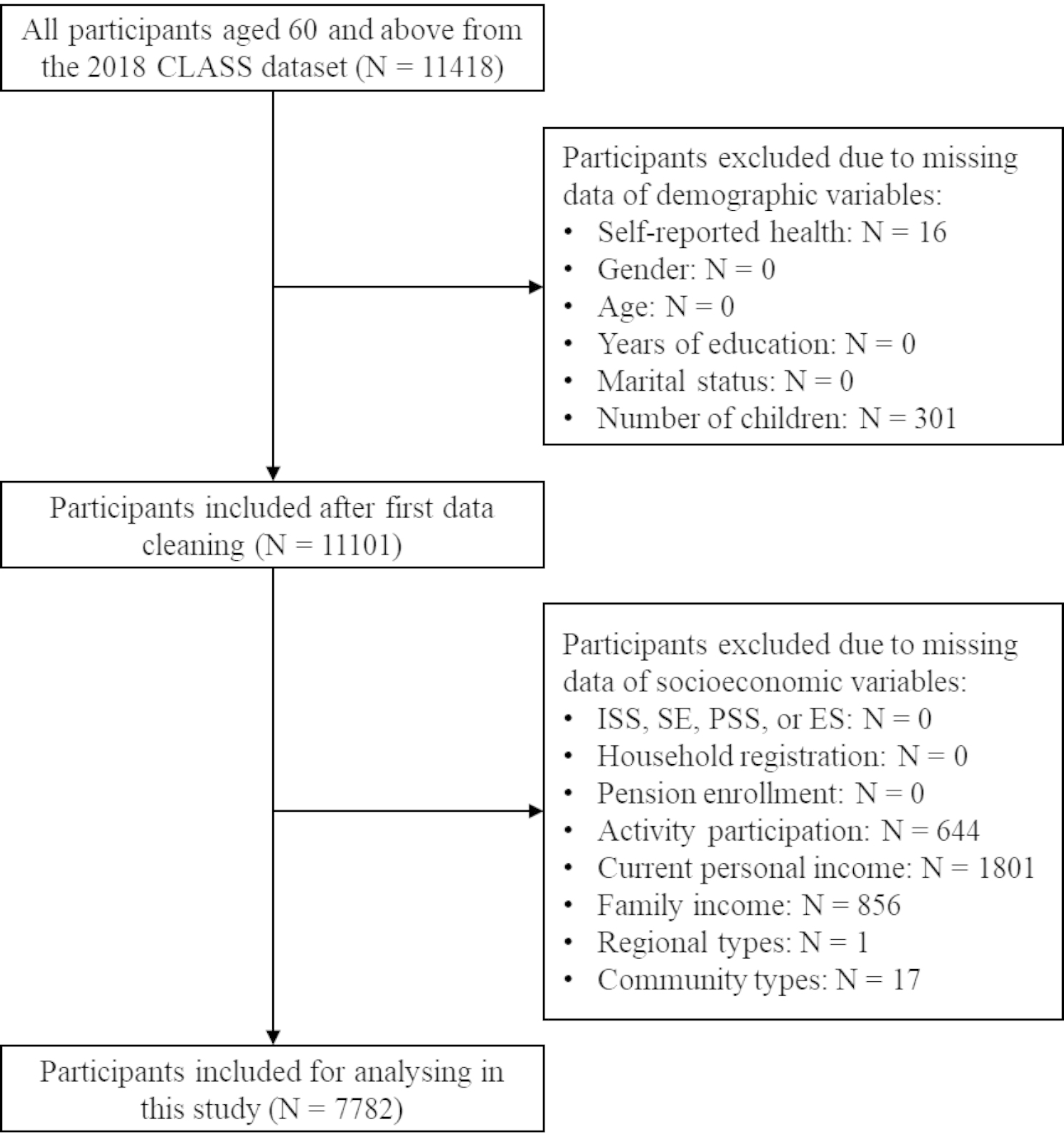
Flowchart of sample selection process

The data used in this study were derived from the 2018 China Longitudinal Aging Social Survey (CLASS), a nationwide and continuous large-scale social tracking survey project carried out by the China Survey and Data Center of Renmin University of China. CLASS uses stratified multi-stage probability sampling to conduct a multi-dimensional survey in 28 provinces in China, aiming to collect high-quality, nationwide, and representative samples to obtain as much as possible demographic, social, and economic information on Chinese residents aged 60 and above for scientific research. As needed, we deleted missing values and outliers from the original data and obtained 7782 observations (Fig. 1).

### Dependent variable: QWB health score

The dependent variable in this study is health status of older adults. To more objectively reflect the health status of older adults and reduce the potential bias caused by subjective perception, based on the questionnaire information in the CLASS database, we refer to the original scale developed by Kaplan et al. [16, 17] and related practices in the existing literature [18], and use the QWB scale as the measure of the health of older adults. The QWB scale is sensitive to changes in disease symptoms and has higher requirements on the design of the questionnaire, which contains four indicators: mobility scale (MOB), physical activity scale (PAC), social activity scale (SAC), and symptom/problem complex (CPX). Furthermore, to avoid the apparent conflict between the weights of the four indicators in the construction of the QWB health score in existing research and the problem that it is out of reality, we attempted to use the AHP method to perform two-stage weighting on the indexes of daily activity (i.e., MOB, PAC, and SAC) and disease symptoms (i.e., CPX), respectively. Hence, combined with the expert consultation method, we have repeatedly solicited and summarized the opinions of clinicians and nurses to assign weights to the measurement items of each indicator, ensuring that the weighting process of the AHP is in line with the actual situation. The QWB score is calculated as follows.

1 $$QWB=1-MOB-PAC-SAC-CPX $$

It should be noted that since some older adults may suffer from multiple chronic diseases or activity disorders simultaneously, the QWB health score may be less than 0. The closer the value is to 1, the better the health is, and vice versa (Table 1).

**Table 1:** AHP weights of QWB scale items based on the CLASS questionnaire

Content	W_1_	W_2_	W	Questionnaire No.
Mobility Scale (MOB)				
Move from bed to bedside chair	0.184	3	0.551	B4-10
Walk around indoors	0.071	3	0.214	B4-11
Go up and down stairs	0.032	3	0.095	B6-1
Has fallen in the past year	0.013	3	0.037	B6-2
Physical Activity Scale (PAC)				
Dress up	0.159	3	0.477	B4-2
Take a bath	0.060	3	0.181	B4-4
Eat independently	0.272	3	0.814	B4-5
Go to the toilet	0.106	3	0.319	B4-9
Lift 5 kg items	0.039	3	0.116	B6-7
Do housework	0.010	3	0.030	B6-9
Social Activity Scale (SAC)				
Walk outside	0.026	3	0.077	B6-3
Take public transport	0.015	3	0.046	B6-4
Go out shopping	0.006	3	0.017	B6-5
Manage money	0.008	3	0.024	B6-6
Symptom/Problem Complexes (CPX)				
Hypertension	0.085	2	0.170	B9-1
Heart disease (such as coronary heart disease, etc.)	0.118	2	0.236	B9-2
Diabetes or elevated blood sugar	0.062	2	0.124	B9-3
Cerebrovascular disease (including stroke)	0.136	2	0.272	B9-4
Urinary system diseases	0.007	2	0.015	B9-13
Glaucoma / cataract	0.005	2	0.010	B9-14
Cancer / malignant tumor (excluding mild skin cancer)	0.221	2	0.441	B9-15

W_1_, W_2_ and W represent the indicator layer weight, criterion layer weight and total weight based on the AHP method. Due to space limitations, only part of the content of CPX is shown

### Independent variable: ISS

The multifaceted construct of ISS concepts and the inconsistency of measurement methods can be complex [19, 20]. The unstable reliability and validity of measurement tools have become the endogenous defects in most studies. To promote the consistent use of well-defined terminology across literature reviews and further discussion in this study, we adopt the classical and widely recognized conception presented by Barrera [10]. After carefully evaluating numerous related concepts, Barrera provided a comprehensive and concise description of the connotation and extension of ISS. He posited that ISS refers to the support individuals can experience from the social field, encompassing three elements: social embeddedness (SE), perceived social support (PSS), and enacted support (ES).

SE refers to the frequency, breadth, or depth of contact with individuals within one’s social networks [10, 21, 22]. As a reasonable measurement item in the questionnaire, we selected the question, “How many friends do you meet or contact at least a month?”. PSS pertains to an individual’s need to build trust with others and achieve a cohesive living environment or atmosphere, as well as their expectations and evaluations of social support [10, 23]. Therefore, we chose “How many friends can you safely discuss private matters with?” as a rational and measurable item for PSS. ES differing from SE and PSS, represents individuals’ substantial mobilization and expression in obtaining solid support from social network members, including emotional support, tangible support, or informational support [10, 22, 24]. We selected the question “How many friends are there to help you when you need it?” as a suitable concept operationalization. The more friends respondents reported in the three questions mentioned above, the greater the SE, PSS, and ES they received, and vice versa. Furthermore, we performed a weighted average of SE, PSS, and ES to obtain a comprehensive measure of ISS using the available information.

### Control variables

To minimize the impact of estimation bias caused by confounding variables in empirical analysis, we considered and included a series of individual and household variables based on research necessity, data availability, and existing literature [25, 26]. the specific control variables, which encompass common demographic characteristics and socioeconomic status, are as follows: gender, age, years of education, marital status, nature of household registration, pension enrollment, current personal income, activity participation, number of children, and family income, among others. (Table 2).

**Table 2:** Descriptive statistics of variables (N = 7782)

Variables	Description	Mean	SD
QWB	Calculated according to the QWB scale and the AHP method	0.778	0.325
SHS	Self-reported subjective health status, “very poor” = 1, “relatively poor” = 2, “fair” = 3, “relatively good” = 4, “very good” = 5	3.376	0.861
ISS	ISS = SE×0.2 + PSS×0.3 + ES×0.5	2.091	1.075
SE	The number of friends respondents could see or communicate with at least once a month, “none” = 0, “only 1” = 1, “only 2” = 2, “3–4” = 3, “5–8” = 4, “9 and more” = 5	2.372	1.277
PSS	The number of friends with whom respondents can communicate or share private matters with confidence, “none” = 0, “only 1” = 1, “only 2” = 2, “3–4” = 3, “5–8” = 4, “9 and more” = 5	2.021	1.139
ES	The number of friends who can provide some help when the respondent is in need, “none” = 0, “only 1” = 1, “only 2” = 2, “3–4” = 3, “5–8” = 4, “9 and more” = 5	2.022	1.231
GEN	“Female” = 0, “Male” = 1	0.508	0.500
AGE	Year surveyed (2018) minus respondents’ birth year	71.189	7.251
EDU	“Not attending school” = 1, “primary school” = 2, “middle school” = 3, “high school/technical secondary school” = 4, “junior college” = 5, “university and above” = 6	2.253	1.042
SPOUSE	“No spouse” = 0, “have a spouse” = 1	0.713	0.453
HUKOU	“Agricultural household registration” = 0, “Non-agricultural household registration” = 1	0.483	0.500
PENS	“None” = 0, “Having participated in Basic endowment insurance for urban employees / endowment insurance for government agencies and institutions / basic endowment insurance for urban and rural residents” = 1	0.794	0.404
PINCO	Total personal income in the past year	8.200	1.412
ACT	The frequency of activity / work in the past week, “never” = 1, “sometimes” = 2, “often” = 3	2.389	0.662
NCHIL	The number of living children	2.5250	1.323
HINCO	The average monthly household income in the past year	9.575	1.254

SD, standard deviation. To mitigate the influence of heteroskedasticity and multicollinearity on the regression results, we perform natural logarithmic transformations on PINCO and HINCO

### Empirical strategy

Considering that the values of the explained variables, the QWB score, have prominent right-censored distribution characteristics, if we use the traditional ordinary least squares regression (OLS) model for analysis, there will be a nonnegligible estimated bias in the research results. Hence, we constructed a high-dimensional fixed-effects (FE) econometric equation and performed regression analysis using the Tobit model. The econometric equation is specified as follows.

2 $$ {QWB}_{i}=\alpha +\beta {ISS}_{i}+\sum _{k=1}^{s}{\delta }_{k}{X}_{ki}+{\phi }_{p}+{\gamma }_{c}+{\tau }_{g}+{\omega }_{f}+{\epsilon }_{i}$$

Where, $$ {QWB}_{i}$$, the dependent variable, is the health score of older adults measured by the QWB scale for individual $$ i$$. $$ \alpha $$ is the constant. $$ {ISS}_{i}$$ is the core independent variable. $$ {X}_{ki}$$ is a set of control variables. $$ {\phi }_{p}$$, $$ {\gamma }_{c}$$, and $$ {\tau }_{g}$$ are provincial FE, regional FE, and community FE, respectively, which control for some unobservable external characteristics such as economic status, social security, medical level, and environmental quality that are related to the health of older adults, whereas $$ {\omega }_{f}$$ is the income FE, which controls occupational factors that may affect the health of older adults (e.g., the original employment mode and labor intensity before retirement). $$ {\epsilon }_{i}$$ is an independent and identically distributed (i.i.d) error term. Data management and analysis were performed using Stata/MP version16.0.

## Results

### Summary statistics

Table 2 provides the descriptive statistical results of the variables involved in this study. The QWB health score of Chinese older adults is relatively high, with a mean of 0.778. In contrast, the subjective self-reported health status of Chinese older adults is relatively poor, with a mean of only 3.376. The means of ISS, SE, PSS, and ES perceived by respondents were only 2.091, 2.372, 2.021, and 2.022, respectively. From the view of control variables, the average age of the participants was 71.189, of which 50.7% were male, 71.3% had spouses, 48.3% had non-agricultural household registrations, and 79.4% had at least one type of pension. Meanwhile, the mean number of children was 2.525; the shares of older adults who had attended no school, primary school, middle school, high school/technical secondary school, junior college, and university and above were 30.5%, 36.6%, 23.2%, 7.6%, 1.8%, and 0.3%, respectively; the means of participants’ income (log) and family income (log) were 8.200 and 9.575, respectively. In addition, the frequency of respondents participating in activities is relatively high, with a mean of 2.389.

### Baseline regression results

In our analysis, we also consider whether multicollinearity exists between the independent variables included in the model, so we conduct a statistical test before estimating the baseline regression model. The test results show that the maximum variance inflation factor (VIF) is 2.92, the minimum VIF is 1.03, and the mean VIF is 1.53, less than the critical value of 10. Hence, we believe there is no need to worry about the potential multicollinearity between variables.

Table 3 reports the estimates of the effect of ISS and its various potential elements on the health of older adults, after adjustment for the evaluated demographic and socioeconomic variables, with coefficients and standard errors, as obtained using the Tobit model. Under the premise of controlling a series of variables, Model 1 also controls the high-dimensional fixed effects of the province, region type, community type, and income mode, of which the estimated results show that ISS has a significant positive effect on the health of older adults (*β* = 0.034, *P* < 0.001). Models 2 to 4 included SE (*β* = 0.024, *P* < 0.001), PSS (*β* = 0.026, *P* < 0.001), and ES (*β* = 0.026, *P* < 0.001) as core independent variables, in which the estimated results showed that each had a highly significant positive and similar effect. When SE, PSS, and ES are included in Model 5 simultaneously, it shows that, although the estimated coefficients have decreased, they are all positive, i.e., greater SE (*β* = 0.013, *P* < 0.009), PSS (*β* = 0.006, *P* = 0.276), and ES (*β* = 0.015, *P* < 0.006) for participants were associated with higher QWB scores, but only SE and ES have statistical significance.

**Table 3:** Baseline regression results

Variables	Tobit 1		Tobit 2		Tobit 3		Tobit 4		Tobit 5
	(1)		(2)		(3)		(4)		(5)
ISS	0.034^***^								
	(0.005)								
SE			0.024^***^						0.013^***^
			(0.004)						(0.005)
PSS					0.026^***^				0.006
					(0.005)				(0.006)
ES							0.026^***^		0.015^***^
							(0.004)		(0.005)
GEN	0.038^***^		0.037^***^		0.038^***^		0.039^***^		0.038^***^
	(0.009)		(0.009)		(0.009)		(0.009)		(0.009)
AGE	-0.011^***^		-0.011^***^		-0.011^***^		-0.011^***^		-0.011^***^
	(0.001)		(0.001)		(0.001)		(0.001)		(0.001)
EDU	0.017^***^		0.017^***^		0.017^***^		0.017^***^		0.017^***^
	(0.006)		(0.006)		(0.006)		(0.006)		(0.006)
SPOUSE	0.032^***^		0.034^***^		0.032^***^		0.032^***^		0.033^***^
	(0.011)		(0.011)		(0.011)		(0.011)		(0.011)
HOKOU	0.004		0.004		0.005		0.007		0.004
	(0.014)		(0.014)		(0.014)		(0.014)		(0.014)
PENS	0.007		0.006		0.009		0.008		0.006
	(0.014)		(0.014)		(0.014)		(0.014)		(0.014)
PINCO	-0.001		-0.001		-0.001		-0.001		-0.001
	(0.006)		(0.006)		(0.006)		(0.006)		(0.006)
ACT	0.061^***^		0.061^***^		0.062^***^		0.061^***^		0.061^***^
	(0.008)		(0.008)		(0.008)		(0.008)		(0.008)
NCHIL	-0.002		-0.002		-0.002		-0.002		-0.002
	(0.005)		(0.005)		(0.005)		(0.005)		(0.005)
HINCO	0.000		0.001		0.000		0.001		0.000
	(0.007)		(0.007)		(0.007)		(0.007)		(0.007)
Constant	1.335^***^		1.358^***^		1.352^***^		1.345^***^		1.336^***^
	(0.090)		(0.091)		(0.090)		(0.090)		(0.090)
Provincial FE	YES		YES		YES		YES		YES
Regional FE	YES		YES		YES		YES		YES
Community FE	YES		YES		YES		YES		YES
Income FE	YES		YES		YES		YES		YES
Pseudo R^2^	0.135		0.133		0.133		0.134		0.135
Observations	7782		7782		7782		7782		7782

FE, fixed effects. Cluster-robust standard errors in parentheses. ^*^, ^**^, and ^***^ indicate statistically significant levels of 0.1, 0.05, and 0.01, respectively

From the view of control variables in Column (1), the signs of all the estimated coefficients conform to the theoretical expectations and reality, which indicated that male participants had higher QWB scores than female participants (*β* = 0.038, *P* < 0.001), and participants who had a spouse had higher QWB scores than those who had no spouse (*β* = 0.032, *P* < 0.003). As for higher level of education (*β* = 0.017, *P* < 0.003) and activity participation (*β* = 0.061, *P* < 0.001), they were positively correlated with QWB scores. Additionally, although the estimated coefficients of urban household registration (*β* = 0.004, *P* = 0.755), pension enrollment (*β* = 0.007, *P* = 0.626), current personal income (*β* = -0.001, *P* = 0.855), and family income (*β* < 0.000, *P* = 0.958), as well as the number of children (*β* = -0.002, *P* = 0.615), conform to the reality in terms of coefficient signs, the associations between them and QWB scores were not statistically significant in Model 1 to Model 5.

### Robustness check

To ensure the reliability of the regression results, in this part, we will use three robustness check strategies to re-estimate the baseline regression model established above. First, change the regression model: Considering that the Tobit model is strictly dependent on data distribution and likelihood function and has strict assumptions, if ${\epsilon }_{i}$ does not follow the normal distribution or does not satisfy the assumption of homoscedasticity, the obtained quasi-maximum likelihood estimation (QMLE) results will be inconsistent. Hence, we use the censored least absolute deviations (CLAD) method with fewer qualifications (only ${\epsilon }_{i}$ is required to be i.i.d) for regression analysis to avoid this problem. Second, replace the dependent variable: (a) use the 25%, 50%, and 75% quantiles of QWB scores as the cut points, and divide the QWB scores into four different levels from low to high; (b) replace the QWB scores with the respondents’ self-reported health scores; these two help us obtain two new ordinal categorical dependent variables that will be estimated through ordered logit and ordered probit model.

From the regression results, as shown in Table 4, the estimated coefficients of ISS in all columns are positive and statistically significant (*P* < 0.001), which reconfirms the positive effect of ISS on the health of older adults and indicates the estimated results in this study are robust.

**Table 4:** Estimated results of robustness check

Variables	Replace Tobit model with CLAD model				Divide the QWB score into four levels				Replace the QWB score with subjective score		
	CLAD 1		CLAD 2		OLogit 1		OProbit 1		OLogit 2		OProbit 2
	(1)		(2)		(3)		(4)		(5)		(6)
ISS	0.014^***^		0.021^***^		0.106^***^		0.062^***^		0.064^***^		0.035^**^
	(0.003)		(0.002)		(0.023)		(0.013)		(0.024)		(0.013)
Control variables	YES		YES		YES		YES		YES		YES
Provincial FE	NO		NO		YES		YES		YES		YES
Regional FE	NO		YES		YES		YES		YES		YES
Community FE	NO		YES		YES		YES		YES		YES
Income FE	NO		YES		YES		YES		YES		YES
Pseudo R^2^	0.027		0.036		0.048		0.050		0.058		0.056
Observations	7749		7540		7578		7578		7782		7782

FE, fixed effects. Standard errors in parentheses, where Columns (1) to (2) report the common standard errors, and Columns (3) to (6) report the cluster-robust standard errors. ^*^, ^**^, and ^***^ indicate statistically significant levels of 0.1, 0.05, and 0.01, respectively

### Endogenous treatment

Although the baseline regression model controls many related demographic and socioeconomic variables and four unobservable heterogeneity factors that do not change over time, there may still be endogeneity problems caused by competing explanations or confounding variables in the research process, which may lead to the bias in regression results. For instance, specific living habits (including diet and exercise) may impact the health status of older adults. However, we have failed to control them effectively due to the lack of corresponding measurement information in the CLASS database. Moreover, whether older adults actively and effectively construct social networks and accumulate social capital in the life course may be affected by individual self-selection behaviors and even the existing social relationships of family members, i.e., the healthier older adults or those with families of higher socioeconomic status may be more conducive to forming interpersonal interactions, communication, and trust from work and life, which in turn can help them obtain much ISS. So, with the help of the propensity score matching (PSM) method, we took the mean of the ISS obtained by older adults as the cut point, set the samples above and below the mean as the treatment (high-ISS) group and the control (low-ISS) group, respectively, and then re-estimated after matching the two groups of samples, which, to some extent, can alleviate the potential endogeneity challenges posed by the problems mentioned above.

Table 5 reports the estimated results using four different matching methods. Regardless of which matching method is used and whether the multiple FEs are controlled or not, ISS has a positive effect on the health of older adults, at least at a 5% significance level, and whose coefficients are very close to the baseline regression results, indicating that, after considering and dealing with potential endogeneity problems, the previous conclusions are still reliable and robust.

**Table 5:** Estimated results based on PSM regression

Matching algorisms	Multiple FE	Treated	Controls	ATT	SE	T-statistic
Nearest neighbor	NO	0.794	0.769	0.026^***^	0.009	3.02
	YES	0.794	0.761	0.033^***^	0.010	3.37
Radius	NO	0.794	0.769	0.025^***^	0.008	3.23
	YES	0.794	0.763	0.031^***^	0.009	3.45
Kernel	NO	0.794	0.768	0.027^***^	0.008	3.44
	YES	0.794	0.763	0.031^***^	0.009	3.50
Local linear regression	NO	0.794	0.773	0.021^**^	0.010	2.03
	YES	0.794	0.765	0.030^**^	0.012	2.45

ATT, the average treatment effect on the treated. SE, standard errors. Multiple FE indicated that we simultaneously control the provincial FE, regional FE, and community FE, as well as the income FE in the econometric model. ^*^, ^**^, and ^***^ indicate statistically significant levels of 0.1, 0.05, and 0.01, respectively

### Mechanism analysis

So far, we have confirmed the relationship between ISS and the health of older adults. Existing literature demonstrates that ISS may positively influence the health of older adults by reducing feelings of loneliness and improving emotional status, ultimately contributing to better physical health [27–30]. To examine whether ISS’s effects on the health of older adults operate through these potential channels, we selected the loneliness and emotional status of older adults, which can be measured through two items in the questionnaire (“Have you felt unaccompanied in the past week?” and “Have you felt in a good mood in the past week?”), as mediating variables for conducting a sequential regression analysis.

From the regression results in Table 6, higher ISS (*β* = -0.081, *P* < 0.001), SE (*β* = -0.071, *P* < 0.001), PSS (*β* = -0.051, *P* < 0.024), and ES (*β* = -0.064, *P* < 0.002) were related to lower loneliness, whereas higher ISS (*β* = 0.220, *P* < 0.001), SE (*β* = 0.159, *P* < 0.001), PSS (*β* = 0.181, *P* < 0.001), and ES (*β* = 0.164, *P* < 0.001) were all associated with a greater positive emotional status. This suggests that ISS and its elements can effectively alleviate loneliness and promote a positive emotional status, thereby improving the health of older adults.

**Table 6:** Estimated results of mechanism analysis based on Ordered Logit model

Variables	Channel 1: alleviating loneliness								Channel 2: improving emotional status						
	OLogit 1	OLogit 2		OLogit 3			OLogit 4		OLogit 5	OLogit 6		OLogit 7		OLogit 8	
	(1)	(2)		(3)			(4)		(5)	(6)		(7)		(8)	
ISS	-0.081^***^								0.220^***^						
	(0.024)								(0.024)						
SE			-0.071^***^								0.159^***^				
			(0.020)								(0.020)				
PSS					-0.051^**^								0.181^***^		
					(0.022)								(0.023)		
ES						-0.064^***^									0.164^***^
						(0.020)									(0.021)
Control variables	YES		YES		YES	YES			YES		YES		YES		YES
Multiple FE	YES		YES		YES	YES			YES		YES		YES		YES
Pseudo R^2^	0.083		0.083		0.083	0.083			0.132		0.131		0.131		0.131
Observations	7635		7635		7635	7635			7635		7635		7635		7635

FE, fixed effects. Cluster-robust standard errors in parentheses. Multiple FE indicated that we simultaneously control the provincial FE, regional FE, and community FE, as well as the income FE in the econometric model. ^*^, ^**^, and ^***^ indicate statistically significant levels of 0.1, 0.05, and 0.01, respectively

### Heterogeneity analysis

In our view, the health promotion effect of ISS may be influenced by different demographic and socioeconomic conditions, which are likely to be heterogeneous. To better identify the manifestations of this heterogeneous effect, we divided total samples into different groups by gender, age, household registration, and personal income and conducted grouping regressions in this part.

According to the analysis in Columns (1) and (2) in Table 7, the regression coefficient in the male group (*β* = 0.036, *P* < 0.001) is larger than that in the female group (*β* = 0.032, *P* < 0.001). The results in Columns (3) and (4) show that, compared with older adults under the age of 80 (*β* = 0.033, *P* < 0.001), the promotion effect of ISS on the health of older adults over the age of 80 (*β* = 0.024, *P* < 0.163) is not statistically significant. Meanwhile, the effect of ISS on health of the rural older adults (*β* = 0.035, *P* < 0.001) is higher than that in the urban group (*β* = 0.032, *P* < 0.001), and the effect of ISS on the health of the high-income older adults (*β* = 0.043, *P* < 0.001) is stronger than that in the low-income group (*β* = 0.027, *P* < 0.001).

**Table 7:** Estimated results of heterogeneity analysis

Variables	Group 1		Group 2		Group 3		Group 4	
	Female	Male	Age $$ <$$ 80	Age $$ \ge $$ 80	Rural	Urban	Low-income	High-income
	(1)	(2)	(3)	(4)	(5)	(6)	(7)	(8)
ISS	0.032^***^	0.036^***^	0.033^***^	0.024	0.035^***^	0.032^***^	0.027^***^	0.043^***^
	(0.008)	(0.007)	(0.005)	(0.017)	(0.007)	(0.008)	(0.007)	(0.008)
Control variables	YES	YES	YES	YES	YES	YES	YES	YES
Multiple FE	YES	YES	YES	YES	YES	YES	YES	YES
Pseudo R^2^	0.137	0.143	0.152	0.085	0.124	0.169	0.140	0.156
Observations	3832	3950	6595	1187	4026	3756	3697	4085

Cluster-robust standard errors in parentheses. Multiple FE indicated that we simultaneously control the provincial FE, regional FE, and community FE, as well as the income FE in the econometric model. ^*^, ^**^, and ^***^ indicate statistically significant levels of 0.1, 0.05, and 0.01, respectively

## Discussion

This study contributes to the emerging literature concerning the relationship between ISS and the health of Chinese older adults using the latest nationally representative sample from China. Based on CLASS data and combined with the AHP method, we constructed QWB scores that can comprehensively assess the health status of older adults and then used econometric methods to conduct a more standardized, rigorous, and robust analysis of the effect of ISS on the health of older adults, including mechanism analysis and heterogeneity analysis, which allowed us to obtain a series of interesting findings in the end.

To the best of our knowledge, most of the existing literature on the effect of ISS on the health of Chinese older adults does not clearly define the concept of ISS and often regards ISS as the relationship and financial support from family members [31, 32]. Thus, we provided a relatively rational measurement of the ISS and its potential elements, which established a common basis for our discussion. In our analysis, we found that greater ISS, including its potential elements (e.g., SE, PSS, and ES), was associated with the better health of older adults, which is consistent with some findings from previous global literature [33, 34]. Substantial evidence from previous studies has demonstrated that people with loneliness and depression who perceive their ISS as poorer have worse health outcomes concerning symptoms, recovery, and physiological functioning [35]. Moreover, we also considered how the health of older adults evolves with ISS, and our findings demonstrated that it may work through alleviating loneliness and improving emotional status. Specifically, ISS has a significant impact on mood, i.e., lack of ISS is the main reason for loneliness among older adults [36], whereas reducing loneliness can improve their living quality and physical function [37]. Conversely, long-term accumulation of loneliness is more likely to lead to an increased risk of all-cause mortality in older adults [38]. Furthermore, positive emotions help individuals get a higher level of healthy cognition [39] and recover faster from the disease status [40], while negative emotions may induce chronic diseases [41].

Additionally, ISS had different health effects among different groups based on gender, age, household registration, and personal income. Our study points out that ISS for male older adults is more likely to improve their health than for female older adults, similar to Natuya Zhuori’s finding [42]. One potential explanation for this gender difference is that men and women may have different social roles and expectations, which can influence their experiences and perceptions of social support [43]. For example, women often take on caregiving responsibilities within families, which can limit their opportunities to engage in social activities and receive ISS [44]. Additionally, the type and quality of social support received by men and women may differ, with men potentially benefiting more from the support received regarding health outcomes [45]. Further research is needed to better understand the underlying factors contributing to these gender differences in the health effects of ISS. For older adults under the age of 80, the health promotion effect of ISS is statistically significant, whereas ISS does not improve the health of older adults over the age of 80. Due to the weakened physical condition and mobility of the high-aged old [46], the opportunities for participation in social activities and social interactions derived from ISS are correspondingly reduced, and the health promotion effect of ISS cannot be effectively realized. We also found that the health status of older adults in rural areas is more sensitive to ISS than that in urban areas, for which a reasonable explanation may be that, compared with urban areas, interpersonal relationships and interaction, as well as exchanges between individuals, are closer and more frequent in rural areas. Thus, rural older adults are more likely to obtain material support and spiritual connection, which is more conducive to promoting individual health. There were similar but interesting findings for income heterogeneity analysis, i.e., ISS has a more significant health promotion effect on high-income older adults, for which a possible explanation might be that high-income older adults commonly have more abundant social resources [47, 48] and can get more ISS accordingly than low-income older adults.

In addition to the above, other findings from our baseline regression results are worth discussing. Male older adults tend to have healthier physical conditions than their female counterparts. Age is a risk factor for the health of older adults. Older adults with higher educational attainment enjoy better health and longer lifespans than their less-educated peers. This result may be explained by the fact that education can improve the socioeconomic status of older adults, help them acquire extensive medical and healthcare knowledge, encourage them to form good and regular living habits, and ensure and maintain their health [49, 50]. Compared to older adults without a spouse, those with a spouse often receive accompanying care and emotional support from their partner, which helps to improve their health [51]. Additionally, older adults can gain physical exercise and emotional relaxation through participating in activities, enriching their lives and consequently improving their health [52]. In contrast, factors such as household registration, pension enrollment, current personal income, the number of children, and family income do not significantly affect the health of older adults. There are several possible explanations for these intriguing results as follows. Although urban older adults are more likely to enjoy convenient and abundant medical and health services than rural counterparts, the relative health advantages of the former may be weakened by factors such as poor environmental quality and high life pressure. As basic endowment insurance in China has been widely implemented among older adults in 2018 and has reached a high level of coverage, participation in endowment insurance does not significantly improve the health of older adults, which does not partially support some previous research [53]. The coefficient of personal income in regression results is negative and not statistically significant, which may be explained by the fact that we used the current income of older adults as a measure of personal economic status. Specifically, on the one hand, high income in old age is generally at the expense of the high-intensity mental or manual labor in the past, which accelerates the depreciation of health capital to a certain extent and indicates that income has a dual effect on the health of older adults [54]; on the other hand, most of older adults in the rural sample generally have lower incomes, which may lead to general poor health performance in the entire sample. Our study also suggested no absolute and significant association between having more children and better health in older adults, which supports evidence from the previous observation [55]. It showed that the more children, the higher cost of raising and caring for offspring in the early stage of older adults, which may also accelerate the depreciation of their health capital. Due to the changes of the times, traditional family structure and intergenerational relations have undergone profound changes, and there has been a phenomenon of children competing to work outside their hometowns or separating from their families due to jobs [56, 57]. To a certain extent, c this reduces the support and care provided by children to older adults, and older adults may not be able to obtain the corresponding pension returns due to the high efforts required to raise their offspring, which is not conducive to the health of older adults. In the end, the regression coefficient of family income is positive but not statistically significant, indicating, to some extent, that the richer the family’s material and economic resources are, the more helpful it is to improve the health status and quality of life of older adults.

These findings in our study have several practical implications that are closely related to the main results and provide specific recommendations for improving older adults’ health status from the perspective of ISS. At present, old-age security and health promotion for older adults in China primarily rely on the government’s formal system and family support. However, as traditional concepts, including filial piety to parents, are changing and family structures and intergenerational relationships are being impacted by modern development [58], greater responsibility is indirectly transferred to the government. It is essential to recognize that in the era of accelerated aging, the government’s pension burden under the constraints of limited resources is getting heavier [59]. Excessive reliance on public health services and material assistance for older adults will inevitably make it difficult to form a comprehensive, sustainable, and high-quality old-age security model for health promotion. As the study has shown the significant positive effects of ISS on the health of older adults, targeted interventions should be developed to enhance ISS and its potential elements (e.g., SE, PSS, and ES), including emotional, practical, and informational support. Policymakers should recognize the importance of the complementary role of ISS to FSS and prioritize developing community-based programs and services that facilitate heart-to-heart conversations, mutual assistance, and intergenerational engagement. This may include creating more accessible public spaces for seniors, organizing social and recreational activities, and promoting volunteer opportunities for the younger generation to engage with older adults. Given our findings on gender, age, and socioeconomic status differences in the health effects of ISS, tailored strategies should be designed to address the specific needs of different older adult groups. For instance, providing more targeted support for men, those under 80 years old, agricultural household registrants, and high-income individuals, who were found to benefit more from ISS in our study. Programs and policies should also consider the unique challenges faced by women, the oldest old, and low-income individuals in accessing ISS and devise interventions that cater to their specific needs. In summary, by recognizing the complementarity of ISS and FSS, as well as addressing other factors affecting the health of older adults, the government, the community, and society as a whole can work together to effectively improve the life and health of older adults.

## Conclusion

The present study was designed to assess the effect of ISS on the health of Chinese elderly, from which the most prominent finding is that ISS, including SE, PSS, and ES, had significant health promotion effects, especially on those who are male, under the age of 80, with agricultural household registration, and with high income. Additionally, our mechanism analysis confirmed that these promotion effects could be reflected through two channels: alleviating loneliness and improving the positive emotional status of older adults. Taken together, these findings suggest a role for government, community, and the whole society in strengthening the supplementary role of ISS to FSS and promoting the effective combination of the two, which, to some extent, will help older adults get health outcomes.

## Data Availability

The data used in this study were all available on the CLASS public website (http://class.ruc.edu.cn/).
